# Swimming-Induced Pulmonary Edema Masquerading as Acute Respiratory Distress Syndrome: A Case Report

**DOI:** 10.7759/cureus.59392

**Published:** 2024-04-30

**Authors:** Madalasa Pokhrel, Nava R Sharma, Saral Lamichhane, Marija Bogojevic, Bolaji Durodola, Adele Gillen, Yorleny Vicioso Mora, Prabal KC, Ashutossh Naaraayan

**Affiliations:** 1 Internal Medicine, Montefiore New Rochelle Hospital, Albert Einstein College of Medicine, New Rochelle, USA; 2 Internal Medicine, Maimonides Medical Center, Brooklyn, USA; 3 Medicine, Manipal College of Medical Science, Pokhara, NPL; 4 Internal Medicine, Gandaki Medical College, Pokhara, NPL; 5 Radiation Oncology, Duke University Medical Center, Durham, USA; 6 Internal Medicine, Rasuwa District Hospital, Kathmandu, NPL

**Keywords:** flash pulmonary edema, ards (adult respiratory distress syndrome), reexpansion pulmonary edema, swimming induced pulmonary edema, sipe

## Abstract

Immersion pulmonary edema, also known as swimming-induced pulmonary edema (SIPE), manifests with cough, dyspnea, hemoptysis, and hypoxemia from flash pulmonary edema after surface swimming, often in healthy young individuals with no predisposing conditions. SIPE commonly resolves spontaneously within 24-48 hours but can be fatal. Post-mortem findings demonstrate heavy, edematous lungs and frothy airways. Although these pathologic findings are like those seen in patients with drowning, SIPE, by definition, is associated with pulmonary edema that develops with a closed glottis without drowning/aspiration. However, patients who develop SIPE during swimming could lose consciousness and drown. Its pathophysiology is poorly understood, and the medical literature infrequently describes SIPE. Due to the multifactorial and complex pathophysiology and the scarcity of medical literature describing SIPE, the diagnosis could be difficult at presentation. This case report elaborates on diagnosing and treating swimming-induced pulmonary edema in a hypertensive and obese female who presented to our emergency room with an acute onset of shortness of breath after recreational swimming in a pool.

## Introduction

Swimming-induced pulmonary edema (SIPE) is a form of pulmonary edema that occurs during water sports activity, usually in young, healthy individuals [1]. It is one of the two types of immersion pulmonary edema, the other being scuba diver’s pulmonary edema (SDPE), both types present with pulmonary edema occurring despite closed glottis and a lack of aspiration. The estimated prevalence of SIPE varies from 1.8 to 60% among swimmer trainees and around 1.4% among triathletes [2,3]. The risk factors for SIPE include systemic or pulmonary hypertension, obesity, cold water immersion, heavy exertion, overhydration, female gender, and wetsuit use [3-6]. Although poorly understood, the basic pathophysiology is central volume redistribution due to increased preload, venous return, and/or stroke volume [1,2]. Typical symptoms of this condition include shortness of breath, chest pain or tightness, cough, and occasional hemoptysis, and it is diagnosed clinically [2].

We present an unusual case of a 57-year-old nonathlete female who developed SIPE after swimming in an indoor swimming pool.

## Case presentation

A 57-year-old female with a history of hypertension and obesity presented to the emergency room with acute-onset shortness of breath after a swim. She was in her usual state of health before swimming. She swam for an hour in the pool at 82 Fahrenheit (27.8 Celsius). After the swim, she walked up to her locker but developed laborious breathing. Besides the shortness of breath, she rapidly turned cyanotic and had an episode of syncope. Bystanders started chest compressions, and after one cycle of cardiopulmonary resuscitation (CPR) and two rescue breaths, they noted a return of circulation and spontaneous recovery of consciousness. Emergency medical services recorded her vitals as blood pressure of 168/122 mmHg, heart rate of 111 per minute, and capillary blood glucose of 111 mg/dl. They started her on the bag and mask ventilation and rushed her to the hospital.

In the emergency room, an initial physical examination showed a Glasgow Coma Scale (GCS) score of 13/15 (E4, V4, M5), a pulse rate of 103 beats per minute, a temperature of 99 degrees Fahrenheit, a blood pressure of 231/120 mmHg, a respiratory rate of 22/minute, oxygen saturation of 100% on a non-rebreather mask providing 100% oxygen at 15 L/min, and a body mass index (BMI) of 36 kg/m^2^. Wet clothes were immediately removed, and she was placed in a warming blanket at 38 Celsius. Physical examination was normal, except for diffuse crackles bilaterally.

**Figure 1:**
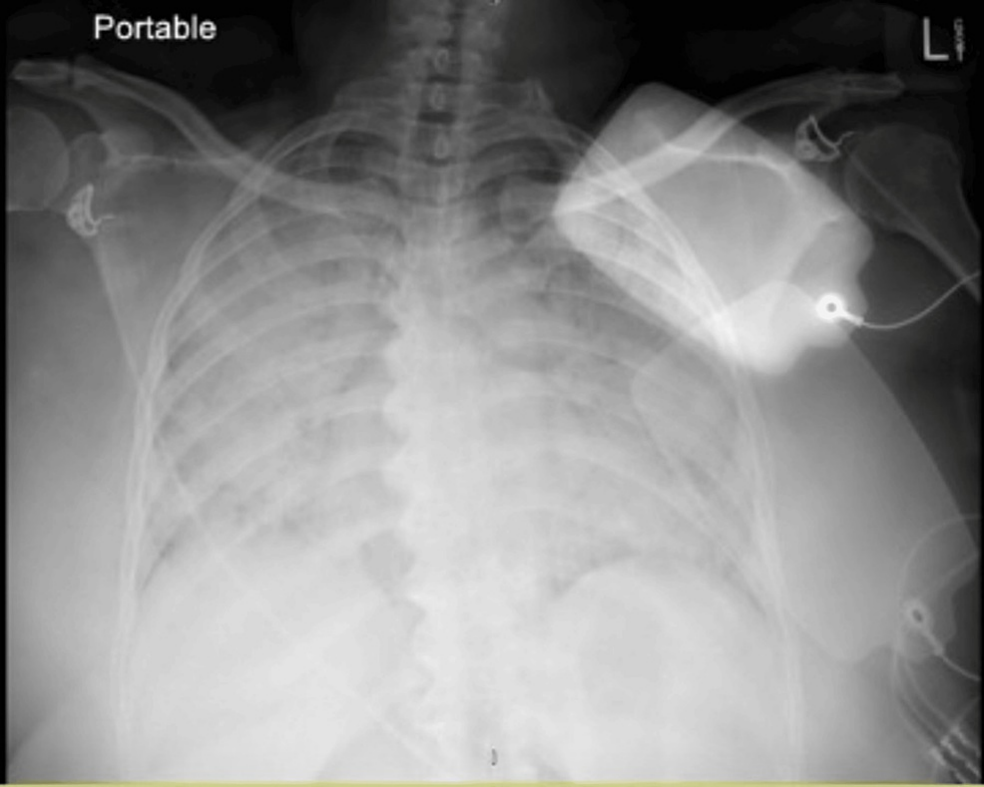
Initial chest X-ray showed bilateral diffuse opacification of the lungs

Blood work revealed severe hypoxemia with a P/F ratio of 70 [arterial oxygen partial pressure (Pao2)/fractional inspired oxygen (Fio2)], as well as anion gap metabolic acidosis caused by lactic acid elevation. Her arterial blood gas parameters were metabolic acidosis with elevated lactate. The patient also had leukocytosis with predominant lymphocytes and transaminitis, as shown in Table 1. The chest X-ray was significant for bilateral diffuse opacification of the lungs (Figure 1). The echocardiogram showed grossly normal left ventricular and right ventricular systolic function with a left ventricular ejection fraction (LVEF) of 55%.

**Table 1:** Laboratory data during initial admission pCO2: partial pressure of carbon dioxide; pH: potential hydrogen

Parameter	Patient's Value	Normal Reference Range
pH	7.009	7.35 - 7.45
pCO2	47 mmHg	35 - 45 mmHg
Bicarbonate (HCO3)	12 mEq/L	22 - 26 mEq/L
pO2	70 mmHg	75 - 100 mmHg
Lactate	15 mg/dL	0.5 - 2.2 mmol/L (4.4 - 19.4 mg/dL)
White blood cell count (WBC)	14,000 cells/mcL	4,500 - 11,000 cells/mcL
Lymphocytes	65%	20 - 40% of total WBC
Aspartate transaminase (AST)	360 U/L	10 - 40 U/L
Alanine aminotransferase (ALT)	276 U/L	7 - 56 U/L

**Figure 2:**
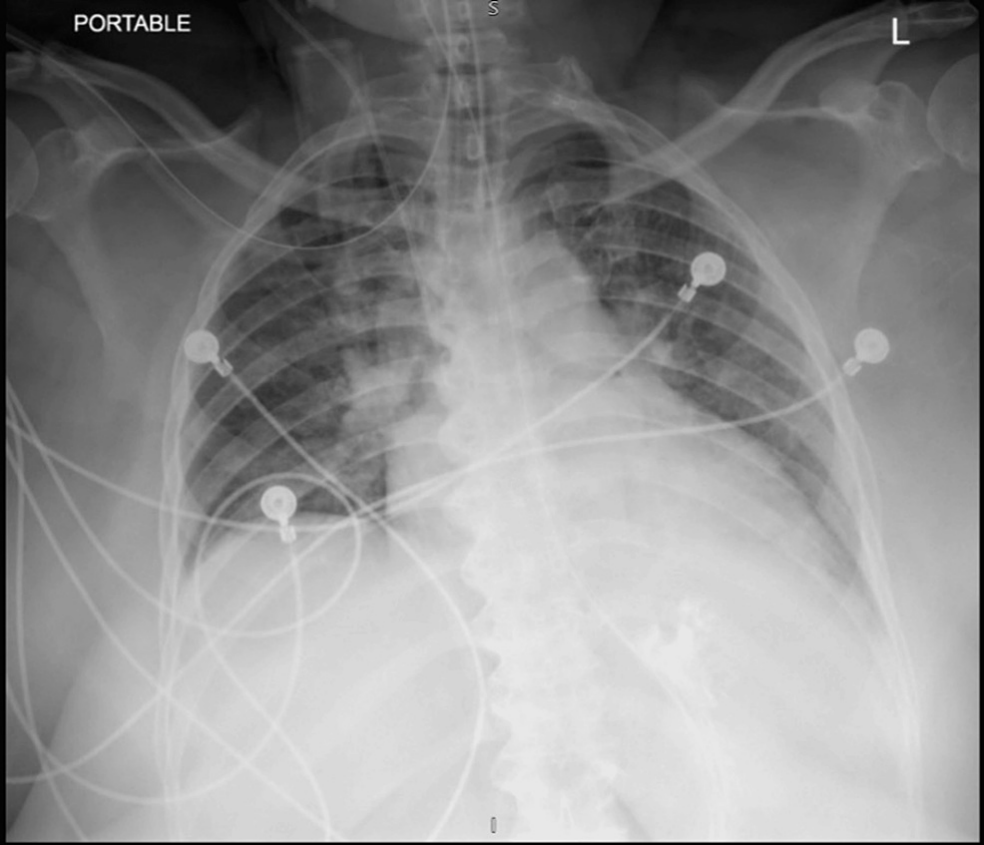
After 48 hours, a chest X-ray with the resolution of diffuse alveolar infiltrates

Intravenous furosemide (120 mg) was immediately given in the emergency room. She was persistently hypoxic and in severe respiratory distress, and thus she was intubated and mechanically ventilated for acute hypoxic respiratory failure. Immediately after intubation, about 100 ml of pink, frothy secretion was suctioned from the patient's airway. Post-intubation, she needed epinephrine and vasopressin for pressure support. She was then transferred to the medical intensive care unit (ICU). The patient was started on prophylactic antibiotics for pneumonia, which were later discontinued after a negative blood culture and infectious workup. After 48 hours of intubation and respiratory support, the chest X-ray showed significant improvement in the resolution of diffuse alveolar infiltrates (Figure 2). She was subsequently successfully extubated on the seventh day of admission.

After a week of ICU management, the patient recovered well and was discharged with proper counseling about avoiding strenuous exercise and swimming. She was followed up in the outpatient unit for further cardiopulmonary workups.

## Discussion

Swimming-induced pulmonary edema is a rare and potentially fatal condition that occurs when healthy swimmers develop flash pulmonary edema during or after an exerting swim without aspirating [7]. Symptoms are those of pulmonary edema, that is, dyspnea (100%), cough (96%), hemoptysis (56%), wheezing (9%), and chest tightness (9%) [8]. Physical examination findings include crackles, rales, and/or wheezing. Hemoptysis may be present. Hypoxia, hypoxemia, and reduced tissue perfusion may finally lead to reduced saturation, lactic acidosis, transaminitis, neurological symptoms, or cardiac hypoperfusion [7]. In our case, the SIPE-induced profound hypoxemia most likely led to cardio-respiratory arrest needing resuscitation. Ludwig et al. suggested the presence of all of the criteria (acute onset of symptoms, hypoxemia, chest imaging consistent with interstitial edema, and no history of specific events) for diagnosing SIPE [9].

Although the pathophysiology of SIPE is not well studied, various studies have found SIPE to be a form of hemodynamic pulmonary edema caused by the redistribution of blood to the heart, leading to an increase in central blood volume [10]. Compressive forces from water immersion and, when applicable, tightly fitting wetsuits on the body increase pressure on the peripheral capacitance vessels, resulting in the central redistribution of blood volume into the thoracic cavity with increased venous return and biventricular preload [10]. This redistribution can increase central venous pressure by 12-18 mmHg and stroke volume by greater than 25% during water immersion at rest [10,11]. In addition to the compressive stress, water also places thermal stress on the body with attendant physiologic effects. Colder water (14°C vs. 32°C) has been shown to cause higher blood pressure and sympathetic tone, characterized by higher norepinephrine levels [11,12].

Furthermore, cold water immersion has been associated with increases in left ventricular end-diastolic volume because of increased peripheral vasoconstriction, and exercise in cold water has also been shown to increase both mean pulmonary artery pressure (MPAP) and pulmonary artery wedge pressure (PAWP) [13]. Even in normal individuals, an acute rise in pulmonary artery wedge pressure (PAWP) above 18-25 mmHg can lead to hydrostatic alveolar edema [13,14]. Elevated hydrostatic pressure leads to multiple-minute breaks in the blood-gas barrier in the lungs, called capillary stress failure, and causes hemoptysis [15,16]. Thus, hydrostatic pulmonary edema coupled with capillary stress fracture provides a plausible mechanism for the development of SIPE in susceptible individuals.

SIPE usually affects people with no underlying health problems and is often misdiagnosed at presentation. Given the physiologic changes involved with immersion, pre-training ingestion of salt tablets and hydration may augment preload and increase the risk of SIPE. Other significant risk factors for SIPE are exertion (as seen in swimmers and triathletes), systemic or pulmonary hypertension, obesity, age, and female gender [3-6]. Lower water temperatures and wetsuits may also contribute to SIPE, as they can increase preload through vasoconstriction [6]. Individuals with an episode of SIPE appear to have a relatively high risk of recurrence (13-75% recurrence rate) [1,17]. Despite all these known risk factors, predicting the susceptibility of individuals to experiencing SIPE is difficult.

The acute onset of classic symptoms after a swim, evident hypoxemia, radiologic evidence of interstitial pulmonary edema, and the rapid resolution of the radiologic pulmonary opacities make SIPE the most likely diagnosis in our case. The absence of aspiration, laryngospasm, left ventricular dysfunction, and confirmed/suspected infection support the diagnosis.

Multiple risk factors like older age, female gender, hypertension, obesity, and more than usual exertion in a novice swimmer out for recreational swimming in an attempt to lose weight were also present. The severity of hypoxemia leading to cardio-respiratory failure, associated shock liver, and acute renal failure added to the severity of the condition and delayed our patient's recovery, delaying extubation despite early clinical and radiologic improvement. Obesity is itself an independent risk factor for delayed extubation [18].

There is a lack of randomized control trials on treatment options for SIPE. Urgent removal from water, placing in a warm environment, and removing wet suits, if any, are recommended. The care is usually supportive, including oxygen therapy, diuretics, and ventilatory support. Off-label use of calcium channel blockers, sildenafil, and other vasodilators with the hopes of counteracting the suspected hydrostatic pulmonary pressures by decreasing pulmonary artery pressures (MPAP and PAWP) has been used with varying success [11,15]. Sildenafil has been shown to reduce mean pulmonary arterial and wedge pressure and has been suggested as a strategy for SIPE prevention [14]. SIPE has a high recurrence rate, so proper counseling of individuals to prevent future episodes is critical. Patient counseling should include the avoidance of swimming in cold water, wet suits, and pre-race salt tablets/hydration. If patients have hypertension, using calcium channel blockers as the first agent could also help prevent SIPE. Patient counseling about the modifiable risk factors is essential to preventing the condition.

## Conclusions

Swimming-induced pulmonary edema (SIPE) is a form of pulmonary edema that occurs during water sports activity, usually in young, healthy individuals. Although the pathophysiology of SIPE is not well studied, it is suspected to be caused by the redistribution of blood to the heart, leading to elevated pulmonary hydrostatic pressures and subsequent injury to the blood-gas barrier, known as capillary stress failure. Individual risk factors include pre-swim salt tablets and hydration, high levels of exertion, systemic or pulmonary hypertension, obesity, age, female gender, and previous occurrence of SIPE. Lower water temperatures and wetsuits may also contribute to SIPE. As participation in endurance sports, including various swimming events and the recreational use of swimming pools, is increasing, physicians should be aware of this rare but potentially life-threatening entity. Although confirming the diagnosis is difficult, SIPE should be suspected in flash non-cardiogenic pulmonary edema after a swim, especially with relevant risk factors, clinical features, and radiological findings. Prompt diagnosis and treatment of SIPE usually result in a good outcome. The evident gap in understanding the pathophysiology and management of SIPE requires further study and research.
